# Dosimetric comparison of MRI-guided and CT-guided intracavitary and interstitial brachytherapy for locally advanced cervical cancer

**DOI:** 10.3389/fonc.2026.1765505

**Published:** 2026-03-04

**Authors:** Wencui Yang, Jun Lu, Li Wang, Haiyun Wang, Peng Li, Qi Zhang

**Affiliations:** 1 Department of Radiotherapy, Xi’an International Medical Center Hospital, Xi’an, Shaanxi, China; 2 Proton Therapy Center, Xi’an International Medical Center Hospital, Xi’an, Shaanxi, China

**Keywords:** cervical cancer, intracavitary and interstitial implantation, MRI imaging, three-dimensional brachytherapy, tumor

## Abstract

**Purpose:**

To compare the differences in dosimetry and toxicities between Magnetic Resonance Imaging (MRI) and Computed Tomography (CT)-guided intracavitary and interstitial implantation for locally advanced cervical cancer, respectively.

**Methods:**

We analyzed 40 cases of locally advanced cervical cancer admitted to our hospital from January 2023 to September 2024. Patients underwent CT-guided intracavitary and interstitial implantation followed by MRI scanning. We compared the volume of HR-CTV and IR-CTV, the dosimetric differences of HR-CTV D_90_ and IR-CTV D_90_, and the dosimetric differences of D_2cc_ for the bladder, rectum, and small intestine under the two localization methods.

**Results:**

The mean HR-CTV and IR-CTV volumes were larger using CT guidance compared with MRI guidance (P<0.05), the differences were statistically significant. The HR-CTV D_90_ and IR-CTV D_90_ were smaller using CT than MRI guidance. The difference was statistically significant (P<0.05). There was a statistically significant difference in the rectum D_2cc_ between CT and MRI guidance (P<0.05), while there was no statistically significant difference for the D_2cc_ of the bladder and small intestine between the two methods (P>0.05).

**Conclusion:**

Intracavitary and interstitial implantation under MRI guidance can significantly improve HR-CTV and IR-CTV D_90_ with reduced target volume and good protection of the rectum, and there is no significant difference for the bladder and small intestine.

## 1 Introduction

Recent global cancer statistics reports indicate that cervical cancer ranks second only to breast cancer among female malignancies, posing a serious threat to women’s health worldwide. In China, the incidence and mortality rates of cervical cancer are even higher, both ranking second (1). Locally advanced cervical cancer (LACC), categorized as stages IB3 to IVA according to the FIGO 2018 staging system, represents a significant global health burden, accounting for a median of 37% of cervical cancer cases worldwide and as high as 90% in resource-limited or lower socioeconomic regions (2). Consequently, the majority of these patients are diagnosed at an advanced stage where the window for radical surgery has already closed, formulating effective non-surgical treatment strategies has become a core challenge in clinical decision-making. The National Comprehensive Cancer Network (NCCN) guidelines recommend concurrent chemoradiotherapy combined with brachytherapy as the standard treatment for locally advanced cervical cancer. Brachytherapy is a radiotherapy technique that places radioactive sources directly inside or near the tumor area, delivering extremely high internal radiation doses to precisely kill cancer cells while maximizing protection of surrounding normal tissues (3, 4). Traditional 2-dimensional (2D) brachytherapy based on plain X-ray films had imprecise dose evaluation and high complication rates (3). In recent years, with continuous innovation in image guidance and treatment equipment, brachytherapy has evolved from traditional 2D irradiation to precise techniques such as 3D brachytherapy and interstitial implantation, significantly improving local tumor control rates. However, currently most domestic institutions can only perform CT-guided 3D brachytherapy. While it can improve tumor target coverage, for cases with large tumors (diameter >4cm), parametrial infiltration, or vaginal invasion, the limited resolution of CT can make it difficult to distinguish tumor boundaries from parametrial and surrounding normal tissues when they are adherent or unclear. This can lead to overly large target volumes resulting in excessive dose to surrounding normal tissues, or insufficient target coverage leading to tumor persistence or recurrence, affecting patients’ long-term quality of life (4). MRI-guided 3D brachytherapy can compensate for this deficiency. MRI offers high soft tissue resolution, clearly displaying the relationship between the tumor and surrounding normal tissues and the tumor location, guiding needle distribution within the tumor region. Theoretically, it allows for more precise contouring and better target coverage. For 3D brachytherapy with interstitial implantation, whether MRI guidance is superior to CT guidance, whether the target volume is more accurate, and whether normal tissue doses are reduced remain subjects of debate (5). Therefore, based on these controversies, our center designed a prospective clinical trial to further compare the dosimetric distribution of CT- and MRI-guided interstitial implantation in locally advanced cervical cancer.

## 2 Materials and methods

### 2.1 Protocol

This prospective study was approved by the institutional review board (IRB) of Xi’an International Medical Center Hospital (2023QN011). All patients provided written informed consent prior to the commencement of the study.

### 2.2 Clinical data

Inclusion Criteria: Detailed pathological data available; classified as stage IIB-IIIC according to the 2018 International Federation of Gynecology and Obstetrics (FIGO) staging system; ECOG performance status score 0-1; no distant metastasis, excluding other diseases; no contraindications to radiotherapy; no significant other organic or psychiatric diseases.

Exclusion Criteria: Cachexia, ECOG performance status score ≥ 2, poor general condition unable to tolerate concurrent chemoradiotherapy; concurrent infection; severe cardiovascular, digestive, urinary, or other systemic diseases; concurrent tumors in other organs; palliative treatment; inability to complete treatment for various reasons.

We included 40 cases of locally advanced cervical cancer admitted to the Department of Radiotherapy, Xi’an International Medical Center Hospital, from January 2023 to September 2024. Patient ages ranged from 39 to 70 years, with a median age of 52 years. Tumor diameters ranged from 2 to 10 cm, with a mean of (4.56 ± 0.16) cm. All cases were histopathologically confirmed as cervical malignancies, including 30 cases of squamous cell carcinoma and 10 cases of adenocarcinoma. Stages were IIB (10 cases), IIIB (25 cases), and IIIC (5 cases).

Risk stratification was performed according to the FIGO 2018 staging system, tumor size, and nodal status, following European Society for Radiotherapy and Oncology (GEC-ESTRO) (9) consensus and institutional protocols. Three risk categories were defined: (1) Low-risk: FIGO stage < IIB with a tumor diameter <4 cm and no lymph node metastasis; (2) Intermediate-risk: FIGO stage IIB–IIIB with a tumor diameter of 4–6 cm and/or limited parametrial involvement; and (3) High-risk: FIGO stage ≥ IIIB with a tumor diameter >6 cm, stage IIIC (positive pelvic or para-aortic lymph nodes), or extensive pelvic sidewall involvement. Accordingly, our cohort consisted of 8 high-risk cases, 30 intermediate-risk cases, and 2 low-risk cases. The sample size of 40 patients was determined based on a convenience sample of consecutive patients meeting the inclusion criteria at our institution within the study period. Although a formal prospective power analysis was not conducted, this cohort provided a total of 80 paired CT and MRI sessions. Given the paired-sample design of this dosimetric study, where each patient served as their own control, this sample size was sufficient to detect statistically significant differences in target volumes and dose-volume histogram (DVH) parameters with a high degree of statistical power. The study was approved by the hospital ethics committee, and all patients provided informed consent (**Table 1**).

**Table 1:** The characteristics of enrolled patients.

Enrolled patients	Level	n (%)
Gender	Female	40 (100%)
Age (years)	Median	52
	Range	39-70
Stage (FIGO 2018)	IIB	10 (25.0%)
	IIIB	25 (62.5%)
	IIIC	5 (12.5%)
Risk Classification	Low	8 (20.0%)
I	Intermediate	30 (75.0%)
	High	2 (5.0%)
histopathologic	SCC	30 (75.0%)
	adenocarcinoma	10 (25.0%)
CRT	non-CRT	0 (0%)
	all-CRT	40 (100.0%)
Period		2023/01/01 ~ 2024/09/30
Performance status	0/1	40 (100%)
	2 or worse	0 (0%)

FIGO, International Federation of Gynecology and Obstetrics; SCC, squamous cell carcinoma; CRT, Concurrent Chemoradiotherapy..

### 2.3 Methods

#### 2.3.1 External beam radiotherapy

All 40 patients underwent pelvic intensity-modulated radiotherapy (IMRT) using a Varian (TrueBeam) linear accelerator with 6MV X-rays. The clinical target volume (CTV) included the entire uterus, the vagina 3 cm below the tumor, and the common iliac, internal iliac, external iliac, obturator, and presacral regions. Our prescribed dose was 50 Gy in 25 fractions, 2 Gy per fraction. The gross tumor volume lymph node (GTVnd) consisted of radiologically enlarged lymph nodes, receiving 60 Gy in 25 fractions, 2.4 Gy per fraction.

#### 2.3.2 Intracavitary combined with interstitial implantation brachytherapy

**Figure 1:**
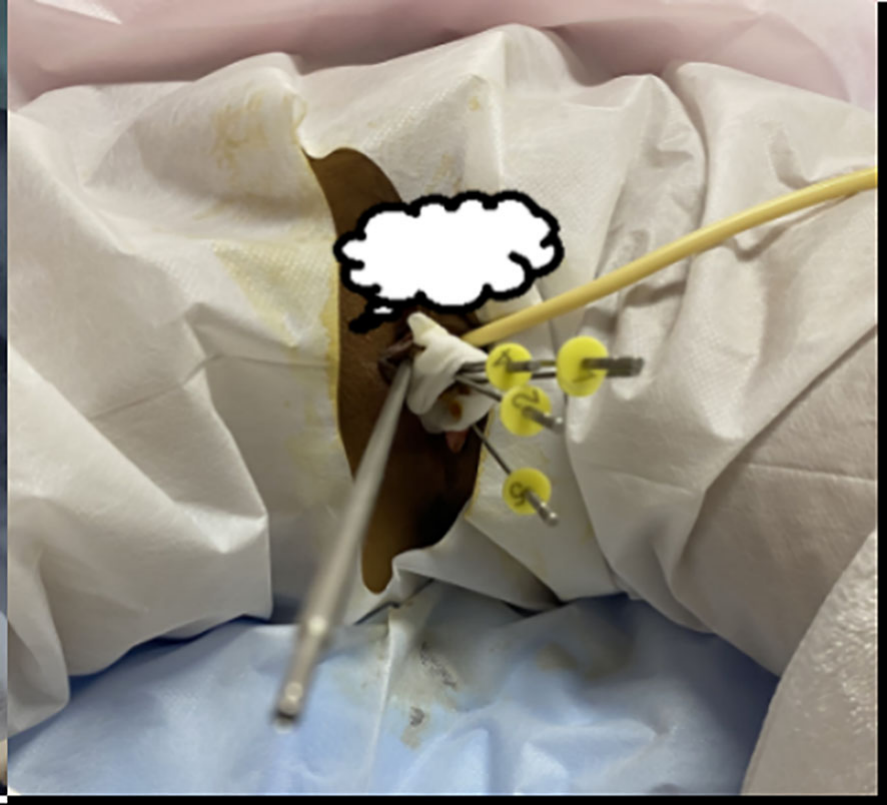
Intracavitary combined with interstitial implantation is performed, with one intrauterine tandem and four interstitial needles placed.

The specific steps were as follows: ① The 24-channel brachytherapy unit of the Variant model is equipped with a range of applicators, including uterine tubes with different angulations (0°, 15°, 30°, and 45°). The appropriate angle is selected based on the curvature of the uterine cavity. Additionally, interstitial needles with a length of 20 cm and a diameter of 1.2 mm are used. Using a Varian Ir192 brachytherapy source, the prescription dose was 6 Gy per fraction, total 4–5 fractions, twice weekly. The first and third fractions involved both CT and MRI localization, total resulting in 80 times CT and 80 times MRI localization sessions for the 40 patients. ② Before implantation, all patients underwent cleaning enema and urinary catheterization. ③ Before brachytherapy, gynecological examination and pelvic MRI scan were performed. Based on pelvic MRI and examination findings, intracavitary tandem and surrounding interstitial needle placement were performed. ④ All patients were placed in lithotomy position. Vaginal packing with lidocaine gauze was used for local anesthesia. The uterine cavity was sounded, and the tandem and interstitial needles were implanted. After completion, the vagina was packed with gauze to diverse the rectum and bladder (**Figure 1**). ⑤ After the insertion of the tandem and interstitial needles, the applicator was securely fixed to the patient’s skin and the treatment couch to prevent any displacement. The patient then underwent CT and MRI scans sequentially. We ensured that the physical position of the applicator and needles remained identical during both imaging sessions. This allowed for a direct dosimetric comparison where the only variable was the imaging modality used for target delineation and plan optimization. ⑥ CT scan localization and MRI scan localization were performed. MRI scans were acquired using a 3.0T scanner (Siemens Skyra) with a pelvic phased-array coil. To ensure optimal target visualization, T2-weighted turbo spin-echo (TSE) sequences were obtained in the sagittal, coronal, and axial planes. The axial slices were oriented perpendicular to the cervical canal or the tandem. The specific MRI parameters were as follows: repetition time (TR) = 3500–4000 ms, echo time (TE) = 80–110 ms, slice thickness = 3.0 mm with no gap, matrix size = 512 × 512, and field of view (FOV) = 200–240 mm. These parameters provided a high in-plane resolution (approx. 0.4–0.5 mm), facilitating the clear differentiation between the tumor, the cervical stroma, and adjacent organs at risk (OARs). ⑦ CT and MRI images were transferred to the Varian Eclipse planning system for fusion. Target volumes and OARs were contoured on both CT and MRI images according to the contouring standards recommended by the Groupe European de Curiethérapie and the GEC-ESTRO (9), including the high-risk clinical target volume (HR-CTV), intermediate-risk clinical target volume (IR-CTV), and OARs.

#### 2.3.3 Quality control for contouring consistency

To ensure the accuracy and consistency of target volume and OARs delineation, all contours were performed following the GEC-ESTRO recommendations and EMBRACE-I/II guidelines. Two senior radiation oncologists specializing in gynecological malignancies (each with >10 years of clinical experience) independently contoured the HR-CTV, IR-CTV, and OARs on both the CT and MRI datasets. Both clinicians were blinded to each other’s contours during the initial phase. A dual-physician review and consensus meeting were then conducted to resolve any discrepancies. If the volumetric discrepancy for HR-CTV exceeded 10%, a third senior chief physician was consulted to determine the final boundary. Furthermore, the image fusion between CT and MRI was strictly verified using the rigid applicator as a reference point to ensure anatomical consistency across different imaging modalities.

#### 2.3.4 Plan optimization and observation indicators

Treatment plans for both CT and MRI guidance were optimized using the Inverse Planning Simulated Annealing (IPSA) algorithm within the Varian Eclipse (v15.6) planning system. The dose calculation was performed using the TG-43 Brachytherapy Formalism. For each fraction, the prescription dose was 6 Gy to the HR-CTV D_90_. Plan optimization was conducted following the EMBRACE II protocol constraints to balance target coverage and OAR sparing. The prioritized dose constraints for optimization were as follows: ① Target coverage: HR-CTV D_90_ ≥ 100% of the prescription dose while maintaining the V_100_≥90%; ② OAR sparing (in EQD2 total dose): Bladder D_2cc_<80 Gy, Rectum D_2cc_<65 Gy, and Small Intestine D_2cc_<70 Gy. During the optimization process, dwell times were adjusted to minimize the dose to OARs without compromising the dose to the HR-CTV, especially when using interstitial needles for eccentric lesions. The same optimization objectives and weightings were applied to both CT- and MRI-based plans to ensure a fair comparison.

### 2.4 Observation indicators

**Figure 2:**
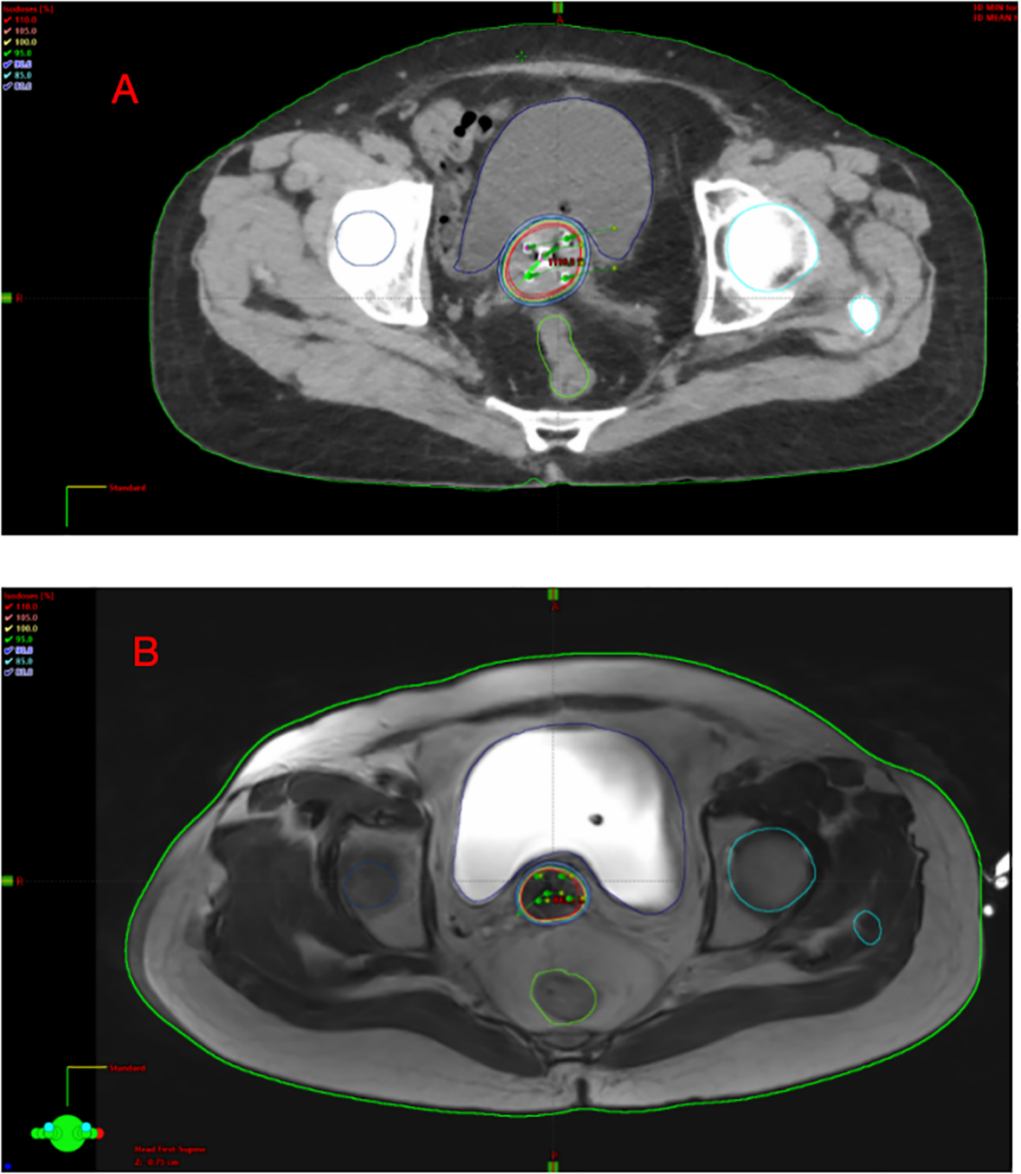
The iso dose of CT scan **(A)** and iso dose of MRI scan **(B)**.

A total of 80 CT and 80 MRI localization sessions were performed. The volumes of HR-CTV and IR-CTV were contoured on images from both modalities. Plans were then designed for both target groups to compare HR-CTV D_90_ and IR-CTV D_90_. The dose differences for the rectum, bladder, and small intestine under the same prescription dose after target volume changes under both methods were also compared. **Figure 2** illustrates the iso dose distributions obtained from CT- (**Figure 2A**) and MRI- guided (**Figure 2B**).

### 2.5 Statistical analysis

Statistical analysis was performed using SPSS 25.0 software. Paired sample t-tests were used for measurement data, described as (mean ± standard deviation). P < 0.05 was considered statistically significant.

## 3 Results

### 3.1 Comparison of target volumes and dosimetric parameters

Detailed comparisons of target volumes and D_90_ values between the two modalities are summarized in **Table 2**. The mean volumes for both HR-CTV and IR-CTV were significantly smaller under MRI guidance compared to CT guidance (p < 0.05). regarding dosimetry, the MRI-guided plans achieved a significantly higher HR-CTV D_90_ compared to CT-based plans (p < 0.05). While the IR-CTV D_90_ was also higher with MRI guidance, this difference did not reach statistical significance.

**Table 2:** Comparison of target volume and dose between the two methods (mean ± SD).

Method (n=40)	HR-CTV (cm³)	IR-CTV (cm³)	HR-CTV D_90_ (Gy)	IR-CTV D_90_ (Gy)
CT guidance	55.05 ±1.16	62.01 ±1.02	5.67 ± 0.18	4.68 ± 2.12
MRI guidance	40.02 ±0.13	45.08 ±0.27	6.21 ± 0.24	4.89 ± 1.14
t-value	5.923	6.091	4.211	2.276
*P*-value	0.002*	0.001*	0.013*	0.098

HR-CTV, High-Risk Clinical Target Volume; IR-CTV, Intermediate-Risk Clinical Target Volume.

D_90_, The dose received by 90% of the volume.

### 3.2 Comparison of OAR doses between the two methods

At a prescribed dose of 6 Gy per fraction, the rectal D_2cc_ was 3.92 ± 0.12 Gy under CT guidance compared to 3.01 ± 1.22 Gy under MRI guidance, with the difference being statistically significant. The bladder D_2cc_ was 4.93 ± 0.35 Gy for CT-guided plans versus 4.71 ± 0.67 Gy for MRI-guided plans, showing a trend toward reduction with MRI guidance, though the difference did not reach statistical significance. For the small intestine (colon), D_2cc_ values were 2.68 ± 0.25 Gy (CT-guided) and 2.49 ± 0.03 Gy (MRI-guided). The D_2cc_ values demonstrated a decreasing trend under MRI guidance, although this reduction did not reach statistical significance. Detailed results are summarized in **Table 3**.

**Table 3:** Comparison of OAR doses between the two methods (Gy, mean ± SD).

Method (n=40)	Rectum D_2cc_	Bladder D_2cc_	Colon D_2cc_
CT guidance	3.92 ± 0.12	4.93 ± 0.35	2.68 ± 0.25
MRI guidance	3.01 ± 1.22	4.71 ± 0.67	2.49 ± 0.03
t-value	4.091	3.120	2.356
*P*-value	0.023*	0.059	0.103

OAR, Organs at risk; D2cc, 2 cm³ volume received the maximum dose.

## 4 Discussion

We investigated the dosimetric differences and toxicities of MRI- and CT-guided intracavitary combined with interstitial implantation for locally advanced cervical cancer. It is well known that the efficacy of radiotherapy for cervical cancer largely depends on brachytherapy. In the era of image-guided adaptive brachytherapy (IGABT), the transition from point-based dosimetry to volume-based parameters has revolutionized the treatment landscape. The landmark EMBRACE-I study, a multicenter prospective trial, has established that MRI-guided IGABT leads to excellent local control (92% at 5 years) and manageable toxicity, setting a new global gold standard (6). With the rapid development of imaging equipment, image-guided 3 dimensional brachytherapy can clearly define target volumes and OARs, precisely displaying target dose, volume, and OAR dose/volume, moving brachytherapy from point doses into the era of volumetric doses, thereby achieving precise treatment. However, the low soft-tissue resolution of CT imaging may lead to ambiguous boundary delineation in patients presenting with extensive target volumes, involvement of adjacent structures such as the bladder or rectum, or infiltration into the parametrium or pelvic sidewall. This can lead to excessive or missed target contouring, creating hot and cold spots, potentially increasing side effects or causing local recurrence/persistence. Furthermore, low resolution CT may lead to overly large target volumes resulting in higher OAR doses, affecting patients’ long-term quality of life. MRI imaging offers the advantage of high soft tissue resolution, clearly displaying tumor size, extent, and boundaries with surrounding tissues, which is a core factor for target contouring in brachytherapy (7). Recent studies utilizing advanced imaging modalities, such as MRI and PET/MRI, have demonstrated that superior soft-tissue resolution and functional assessment are critical for distinguishing residual tumor from post-EBRT changes, thereby preventing unnecessary irradiation of healthy tissue and improving long-term treatment outcomes (8). MRI-guided source placement and tumor target delineation offer superior soft-tissue resolution and enhanced differentiation between malignant and adjacent normal tissues, thereby achieving more precise dose delivery to the target volume. This can improve local tumor control rates, effectively reduce damage to surrounding tissues like the bladder and rectum, lower the incidence of complications such as radiation cystitis and proctitis, and improve patient long-term quality of life (9, 10).

For patients with eccentric tumors or parametrial invasion reaching the pelvic sidewall, this study employed intracavitary combined with interstitial implantation therapy. CT-localized target contouring and MRI-localized target contouring were performed separately and then fused. During brachytherapy target contouring, MRI clearly displays cervical tumors, parametrial tissues, and tumor boundaries with the bladder/rectum. CT images show cervical boundaries more vaguely, with less clear tumor display, potentially leading to overestimation of HR-CTV. This can result in larger HR-CTV volumes, higher OAR doses, and greater side effects. The GEC-ESTRO and ESTRO recommend using DVH parameters to describe target and OAR doses (11). Targets are described starting with low doses using D_90_ for GTV, HR-CTV, IR-CTV (D_90_ refers to the dose received by 90% of the volume). This study used HR-CTV D_90_ and IR-CTV D_90_. For OARs, the maximum dose to the smallest volume receiving the highest dose (e.g., 2 cm³, 1 cm³) is defined as D_2cc_, D_1cc_ (12). Potter et al. (13) retrospectively compared MRI/CT-guided 3 dimensional brachytherapy, showing that MRI-guided 3 dimensional brachytherapy achieved high complete response rates, with 3-year local control rates up to 95%, significantly prolonging patient survival. Viswanathan et al. (14) compared HR-CTV and IR-CTV between CT and MRI guidance, finding that MRI-guided HR-CTV and IR-CTV were significantly smaller than CT-guided volumes. The present study demonstrated that MRI-contoured HR-CTV and IR-CTV volumes were significantly smaller compared to those contoured on CT images (p < 0.05), which aligns with findings from prior studies (13–15). This difference is attributed to the superior soft-tissue resolution of MRI, which enables clear visualization of cervical tumors, parametrial structures, and tumor interfaces with the bladder and rectum, thereby facilitating more accurate contouring. In contrast, the limited soft-tissue resolution of CT obscures the boundaries between tumor and adjacent normal tissues, often leading to overestimation of target volumes.

GEC-ESTRO recommendations (11) and DVH dose-effect relationships require HR-CTV D_90_ doses of at least 85 Gy to achieve local control rates above 90%. For large tumors, HR-CTV D_90_ doses should reach at least 87 Gy. Failure to achieve >87 Gy due to applicator or planning design reasons can lead to local persistence (15). This study showed that MRI-guided HR-CTV and IR-CTV volumes were 40.02 ± 0.13 cm³ and 45.08 ± 0.27 cm³, respectively, while CT-guided volumes were 55.05 ± 1.16 cm³ and 62.01 ± 1.02 cm³, respectively. MRI-defined target volumes were significantly smaller than those delineated under CT guidance (p < 0.05). The analysis suggested that low resolution CT maked it difficult to distinguish between cervix, corpus uteri, vagina, and parametrial invasion areas. To avoid missing lesions, suspicious areas were included in HR-CTV, leading to larger targets. MRI provides clear visualization of tumor tissue, parametrial lesions, and surrounding normal anatomy, enabling precise delineation of HR-CTV. This enhanced anatomical discrimination, supported by clinical correlation, constitutes a principal factor contributing to the consistently smaller target volumes achieved with MRI guidance compared to CT-based contouring. The results of this study showed that CT-guided HR-CTV and IR-CTV D_90_ were 5.67 ± 0.18 Gy and 4.68 ± 2.12 Gy, respectively, while MRI-guided values were 6.21 ± 0.24 Gy and 4.89 ± 1.14 Gy, respectively. CT-guided doses were lower than MRI-guided doses, and the difference in HR-CTV D_90_ was statistically significant (p < 0.05). The reason is considered to be that this study used intracavitary combined with interstitial implantation, distributing needles into tumor tissue and parametrial areas, overcoming the limitations of traditional three-channel dosing, providing local boost to eccentric tumors. Additionally, MRI guidance clearly distinguishes tumor tissue, allowing precise needle placement in tumor areas, further improving target coverage, hence higher HR-CTV and IR-CTV D_90_ doses compared to CT guidance.

Based on European recommendations (9) for bladder, rectum, and small intestine D_2cc_, rectum and small intestine D_2cc_ should be less than 70–75 Gy, and bladder D_2cc_ less than 85–90 Gy. This study’s results showed that all cases under CT or MRI had lower doses than similar studies, yet local control rates did not significantly decrease. This study showed that CT-guided rectum D_2cc_ dose was 3.92 ± 0.12 Gy, while MRI-guided was 3.01 ± 1.22 Gy. MRI-guided rectum D_2cc_ was significantly lower than CT-guided, with statistical significance (P < 0.05). Recent evidence from the retro-EMBRACE and EMBRACE-I cohorts indicates that the risk of major rectal toxicity increases significantly when the rectal D_2cc_ exceeds 65–75 Gy (EQD2) (16). By accurately identifying the interface between the posterior cervix and the rectum on MRI, we were able to minimize the dose to the rectal wall. MRI-guided bladder and small intestine D_2cc_ showed a decreasing trend but no statistical significance. This discrepancy is likely attributable to the consistent anatomical presentation and minimal volumetric variation of the bladder between MRI and CT imaging modalities, thereby yielding no statistically significant dosimetric difference. Furthermore, the spatial separation between the small intestine and the HR-CTV, coupled with the sufficient soft-tissue contrast for intestinal delineation on CT, results in only marginal variations in dose metrics.

However, this study has several limitations. First, it utilized a convenience sample from a single center, which may limit the generalizability of the findings and the statistical power for certain dose-volume metrics. Second, the current report focuses exclusively on dosimetric comparisons; given that patient enrollment concluded in September 2024, long-term clinical follow-up data regarding local control and late-term toxicities are not yet available. Third, the implantation procedure was performed under local anesthesia, which occasionally led to prolonged procedure times due to patient discomfort, potentially affecting the optimal distribution of interstitial needles. The future implementation of general anesthesia may further enhance procedural precision and patient quality of life. Despite these limitations, this study demonstrates that MRI-guided intracavitary and interstitial brachytherapy for locally advanced cervical cancer enables significantly more precise target delineation, resulting in smaller target volumes and superior dose coverage (D_90_) compared to CT-based planning.

## 5 Conclusion

In conclusion, this study demonstrates that MRI-based planning for intracavitary and interstitial brachytherapy offers significant dosimetric advantages over CT-based planning in locally advanced cervical cancer. By leveraging superior soft-tissue resolution, MRI enables more precise target delineation, resulting in significantly reduced HR-CTV volumes and enhanced target coverage (D_90_). These findings support the routine use of MRI guidance to maximize the therapeutic ratio and potentially improve long-term clinical outcomes.

## Data Availability

The original contributions presented in the study are included in the article/supplementary material. Further inquiries can be directed to the corresponding authors.
